# Unveiling ableism and disablism in assessment: a critical analysis of disabled students’ experiences of assessment and assessment accommodations

**DOI:** 10.1007/s10734-022-00857-1

**Published:** 2022-05-07

**Authors:** Juuso Henrik Nieminen

**Affiliations:** Faculty of Education, The University of Hong Kong, Hong Kong, SAR, China

**Keywords:** Assessment, Assessment accommodations, Ableism, Disabilities, Inclusion

## Abstract

This study examines the underlying mechanisms of ableism and disablism in the assessment of student learning in higher education. Globally, higher education institutions rely strongly on assessment accommodations (e.g., extra time in tests) to ensure disabled students’ participation in assessment. This is also the case in Finland. Even though research on disabled students’ experiences of assessment has repeatedly shown that both assessment and assessment accommodations cause barriers for disabled students’ inclusion, critically oriented research on this topic has been scarce. In this study, the frameworks of ableism and disablism are used to unveil how assessment is predominantly designed for “the ideal, able student” and how disabled students are framed as “the Other” through assessment. This work is based on an analysis of 139 disabled students’ experiences of assessment and assessment accommodations as collected through an open-ended, institution-wide survey at a Finnish university. The findings reveal the profound role of assessment in excluding and marginalizing disabled students as unfit to take part in the testing cultures of academia. The accommodation model is shown to hold disabled people responsible for their own exclusion. Disablism is identified in students’ experiences of outright discrimination, such as teachers denying access to assessment accommodations when they are officially granted. This study offers a novel, critical means of discussing assessment from the viewpoints of diversity and inclusion. It also proposes future trajectories for anti-ableist assessment approaches that understand diversity as enriching, rather than obscuring, assessment.

## Introduction

As higher education has shifted from an elitist institution to fostering social inclusion for all (the Universal Declaration of Human Rights; United Nations, 1948), legislation in many countries has started to promote wider access to higher education for disabled people.^1^ Yet, despite efforts to promote access, profound barriers for inclusion remain within the “ableist ivory towers” of academia (Mellifont, 2021). As the physical spaces and teaching practices of academia were not originally designed with student diversity in mind, many inaccessible practices hinder disabled students’ full inclusion (Dolmage, 2017).

Assessment is a key example of such inaccessible design. Assessment has struggled to deal with the increasing diversity of students in higher education: after all, its predominant purpose is to reveal and certify differences rather than to celebrate them (Ketterlin-Geller & Johnstone, 2006). In fact, assessment is deemed so inaccessible that assessment accommodations (e.g., extra time in exams) are administered in higher education institutions around the world. Accommodations are presented as a fair way to promote inclusion as they enable disabled students a fair opportunity to excel in assessment (Weis & Beauchemin, 2020). While this goal seems warm-hearted, the overreliance on accommodations reveals how, in higher education, disabilities are predominantly understood through *the medical model* *of disability* which frames disability as a personal deficit that needs to be accommodated, cured, and fixed (Haegele & Hodge, 2016; Liasidou, 2014). Disabilities are then framed as obscuring the results of assessment and challenging its validity through “construct-irrelevant variance” (Ketterlin-Geller & Johnstone, 2006, p. 165).

Another way to understand disabledness is the *social model of disability* which emphasizes that disabilities are constructed in their socio-cultural and socio-historical contexts (Haegele & Hodge, 2016; Riddell & Weedon, 2006). This model sheds light on how inaccessible practices *disable* people. The social model has guided accessible assessment design that seeks to “design out” barriers and thus reduce the need for accommodations. This is achieved through accessible design that aims for diverse, flexible, and relational assessment (e.g., Universal Design for Assessment; Ketterlin-Geller & Johnstone, 2006; Tai et al., 2021; Waterfield et al., 2006). Both approaches—individual accommodations and inclusive design—have been introduced as a way to promote inclusion in assessment. Yet, it is the medical model that dominates both assessment practice and research (Nieminen, 2021; Tai et al., 2021). For example, accommodations are prevalently mandated in legislation, while students’ right to inclusive and accessible assessment design is not. Researchers have called for inclusive assessment design for decades (see, e.g., Brandt, 2011; Edwards et al., 2022; Hanafin et al., 2007; Madriaga & Goodley, 2010; Redpath et al., 2013). The accommodation model has been characterized as “antithetical to the principles of an inclusive discourse” (Liasidou, 2014, p. 124), causing “profound, encompassing ideational, practical and social justice concerns” (Hanafin et al., 2007, p. 445). Even then, inclusive assessment design remains in margins of higher education and the accommodation model prevails.

How disabled students’ learning is assessed relates to the overall role of assessment in higher education. There has been a prevalent argument that assessment should not only certify students’ skills but also to support learning and promote lifelong learning skills (Boud & Soler, 2016). In practice, assessment stagnates as testing characterizes academia and student-centered assessment practices are “taken up slowly, if at all” (Boud et al., 2018, p. 1107). This is partly due to the increasing class sizes in mass higher education (Williams et al., 2014). However, assessment has an even deeper purpose that is not based on learning but on maintaining the meritocratic underpinnings of higher education. Torrance (2017) situated assessment in its broader context of neoliberal governance to analyze how educational assessment has become a vehicle to promote the values of competition and individualization. Such discourses might be especially strong in higher education, where exams, scores, and grades are used as “objective” measurements of academic standards:

Assessment plays a very important part in underpinning the discourse of meritocracy in higher education, which maintains that the ‘best’ students gain access to the ‘best’ universities, and subsequently, on the basis of their degree classification, move into the most prestigious and highly remunerated areas of employment. Upholding the idea of inviolable academic standards is critical to maintaining the public legitimacy of this system of selection. (Riddell & Weedon, 2006, p. 57)

Tensions arise as mass higher education widens the diversity of students *and* maintains its exam-driven assessment culture. This is how testing and the medical model of disability justify each other: exams produce objective and comparable data of “learning outcomes,” and accommodations are then needed to provide disadvantaged students a fair access to exams. This way the validity of assessment is ensured for the purposes of maintaining the meritocracy, as Riddell and Weedon (2006) note. Unsurprisingly, then, academic support for disabled students is often abridged to exam-focused accommodations in such test-driven environments (see, e.g., Nieminen, 2021).^2^

In this study, rather than understanding “inclusion” as a procedural mechanism to ensure participation in tests through accommodations, or as a matter of accessible assessment design, I discuss the politics of inclusion by examining the contribution of assessment to the exclusion and marginalization of disabled students. I analyze assessment through the frameworks of ableism and disablism (Dolmage, 2017; Goodley, 2014) to unveil how assessment is designed for the ideal, “normal” student, and how assessment frames disabled students as “the Others,” as related to the ideal student. These issues are brought to life through an analysis of 139 disabled students’ experiences of assessment and assessment accommodations based on an open-ended survey study. The lenses of ableism and disablism are used to show how test-driven assessment and the accompanied accommodation model present a major mechanism for preventing the full inclusion of disabled students in academic spaces. The study is situated in the Finnish context that presents a low-stakes assessment culture and high teacher autonomy: in this context assessment *could* quite conceivably be used for the purpose of radical inclusion.

## Disabled students’ experiences of assessment and assessment accommodations

Overall, disabled students’ lived experiences have remained in the margins of assessment research. Psychological approaches dominate the field (Nieminen, 2021). For example, studies have commonly sought to identify which accommodations are effective for which disability types (cf. the medical model of disability) (e.g., Weis & Beauchemin, 2020). Qualitative studies have also drawn predominantly on the medical model, focusing on students’ “disorders,” “symptoms,” and “deficits” rather than on social dimensions of disability (e.g., Ofiesh et al., 2015; Slaughter et al., 2020).

While disabled students’ experiences of higher education studies and adjustments have been studied extensively, fewer studies have focused on assessment and assessment-specific accommodations. Assessment-focused literature mostly concerns the barriers caused by timed closed-book examinations, and this literature tends to paint a negative picture. Disabled students have largely reported that exams create barriers for their learning and inclusion. This seems to be a global issue: such findings have been reported in, for example, Zimbabwe (Govero & Govero, 2019), Croatia (Babic & Dowling, 2015), Spain (Lopez-Gavira & Moriña, 2015), Greece (Stampoltzis & Polychronopoulou, 2009), Finland (Nieminen, 2020), the UK (Fuller et al., 2004a, 2004b; Riddell & Weedon, 2006), the USA (Denhart, 2008), and Australia (Edwards et al., 2022; Ryan, 2007). Because of the inaccessibility related to their format, exams have been found to cause issues for students with dyslexia (Nieminen, 2020; Riddell & Weedon, 2006; Stampoltzis & Polychronopoulou, 2009), autism and Asperger’s syndrome (Madriaga & Goodley, 2010), ADHD (Jansen et al., 2017), chronic illnesses (Kendall, 2016; Magnus & Tossebro, 2014; Majoko, 2018), sensory and mobility impairments (Hopkins, 2011), and for students who have hearing and vision impairments (Kendall, 2016; Redpath et al., 2013). Brandt (2011) even raised “exam phobia” as a type of “impairment” (p. 111). It has been noted that disabled students choose their courses in order to avoid exams (Fuller, Bradley, et al., 2004; Lopez-Gavira & Moriña, 2015), which in turn might hinder their studies (Magnus & Tossebro, 2014). According to students’ own experiences, exam-driven assessment cultures rarely allow disabled students to show the full spectrum of their diverse skills and abilities (Kendall, 2016; Ryan, 2007; Stampoltzis & Polychronopoulou, 2009).

When it comes to disabled students’ experiences of student-centered forms of assessment (e.g., self- and peer-assessment, portfolios), literature is scarce. Recent contributions have hinted that accessible design, flexibility, and choice of assessment practices benefit disabled students’ learning (e.g., Morris et al., 2019; Tai et al., 2021). However, how alternative forms of assessment could support *inclusion* is still largely an unanswered question (Nieminen, 2022). Ashworth and colleagues (Ashworth et al., 2010) offer an important contribution in this front, studying student-centered assessment in performing arts. In arts, it was possible to use alternative forms of assessment, as the overall idea of “academic standards” was open for discussion. Assessment could then rely on relational practices rather than on testing and measurement, which enabled the participation of students with complex disabilities.

Earlier research on disabled students’ experiences of assessment accommodations is nuanced. Overall, accommodations support disabled students’ performance in assessment (e.g., Denhart, 2008; Kendall, 2016). At the same time, multiple shortcomings have been noted. First, the process of acquiring accommodations can be resource-taking. One must first acquire a diagnosis which in itself can be expensive and time-consuming, and then find the “path of least resistance” to succeed in academia (Hopkins, 2011). As Goode (2007) put it, accessing assessment accommodations often requires disabled students to battle the system: “These aren’t issues other students have to deal with” (p. 46). Moreover, unclear instructions about how to apply for accommodations hinder students from receiving the support they need (Korkeamäki & Vuorento, 2021). Because of the *hassle*, many disabled students do not apply for assessment accommodations (e.g., Nieminen, 2020). Even when they do, the process forces students to disclose their disabilities, often to multiple stakeholders and repeatedly over one’s studies. Because of these reasons, many disabled students find disclosure to be shameful and stigmatizing (Madriaga & Goodley, 2010; Magnus & Tossebro, 2014; Marshak et al., 2010; Stampoltzis & Polychronopoulou, 2009). Furthermore, issues have been raised about the stigmatizing nature of accommodations as they frame disabled students as “abnormal” and indeed “unable,” often visibly in front of others (Hanafin et al., 2007; Redpath et al., 2013). For example, it has been reported multiple times how, when the exam is over, everyone else leaves the exam hall except those with extra time (e.g., Goode, 2007; Nieminen, 2020). Finally, support offered for disabled students is often abridged merely to test accommodations (Brandt, 2011; Babic & Dowling, 2015). For example, Waterfield and colleagues (2006) noted that disabled students received accommodations for exams but not for other assessment practices, such as portfolios and essays.

Critical theories are notably absent from earlier analyses. Psychological approaches have neglected the social, cultural and political aspects of disability, focusing on students’ deficits and impairments and how those could be overcome through accommodations (e.g., Ofiesh et al., 2015; Slaughter et al., 2020). In contrast, social model-oriented studies have addressed some social aspects of disabledness, though largely through inductive, data-driven analyses (e.g., Brandt, 2011; Edwards et al., 2022; Hanafin et al., 2007; Madriaga & Goodley, 2010). I argue that this has resulted in similar reports of negative assessment experiences over and over again, hindering us from understanding the systemic forms of exclusion and discrimination which underlie such “negative experiences.” As Goodley (2014) notes, there has never been a greater need for *theory* amidst the dominant approaches of atheoretical empiricism in educational research—and such approaches seem to dominate earlier research on assessment of disabled students in higher education.

## Theoretical framework: ableism and disablism

Ableism refers to the ideology of valuing abilities and abledness over disabilities and disabledness (Baglieri & Lalvani, 2019; Campbell, 2009). Ableist practices govern bodies and minds towards “normal” and “able.” Similar to other “isms,” ableism is a broad concept that manifests in practice in its socio-historical contexts (Campbell, 2009). Dolmage (2017), who has conceptualized academic ableism, defines the term as follows:

Ableism, on the other hand, instead of situating disability as bad and focusing on that stigma, positively values able-bodiedness. In fact, ableism makes able-bodiedness and able-mindedness compulsory. [...] Ableism renders disability as abject, invisible, disposable, less than human, while able-bodiedness is represented as at once ideal, normal, and the mean or default. (Dolmage, 2017, p. 7)

The power of ableism is seen in how it provides information about bodies and minds, and how it steers people to understand themselves through certain kinds of knowledge. The concept of *normalization* considers how people learn to analyze themselves against “the normal” (Foucault, 1977). Ableist practices and structures indeed steer people toward the ideal normal (Dolmage, 2017). The valued modern citizen is cognitively able, and thus normal (Goodley, 2014). As Foucault (1977) reminded, normality is never neutral, but deeply political. Goodley (2014) discusses normalization in modern societies where people are steered toward “the neoliberal self [as] an able-bodied entrepreneurial entity” (p. 29). Neoliberalism strives for competition and performativity, being a political ideology that actively hides structural inequalities (Dolmage, 2017). Neoliberalism frames assessment as a competitive ranking process which renders academic skills and knowledge into market commodities: grades and degrees (Nieminen, 2021; Torrance, 2017).

Ableist practices teach us to understand ourselves through our bodily and cognitive abilities and to value them accordingly (Baglieri & Lalvani, 2019; Dolmage, 2017). This is conducted through normalizing practices through which students come to understand themselves in relation to a certain idea of normal abilities. Historically, disabled people have been largely excluded completely from higher education. Yet normalization is not always based in force; students and teachers take willingly part in normalizing practices (Goodley, 2014). Ableism is seen as teachers and researchers aim to normalize and “cure” disabilities rather than valuing their uniqueness. Importantly, normalizing practices internalize the project of ableism. The concept of internalized ableism refers to how disabled people learn to view themselves as lesser, separating themselves ontically from “the normal” and from “others” as being different, special, and unsuitable (Campbell, 2009).

Another useful concept for understanding how ableism operates in assessment is the one of *dividing practices* (Foucault, 1982). Dividing practices produce knowledge about how people can be classified and categorized. These practices “categorize, classify, distribute, and manipulate subjects who are initially drawn from a rather undifferentiated mass of people” (Tremain, 2017, p. 55). Through dividing practices, people learn to understand them in relation to their own and others’ categorizations. In disability-related work, it has been noted that dividing practices maintain the ontological separation of the normal from the pathological (Campbell, 2009), often through inaccessible design that divides the population into disabled and abled (Tremain, 2017). For example, if one wishes to access assessment accommodations, one needs to have a diagnosis, which itself is a powerful dividing practice: only through the category of “disabled” can one access assessment accommodations.

Ableism affects everyone through its normalizing gaze. Only a few of us fit the normative ideal of the able, productive citizen throughout our lives. However, only disabled people are discriminated against through direct forms of ableism, *disablism* (Baglieri & Lalvani, 2019; Goodley, 2014). Disablism is a set of practices that “promotes the differential or unequal treatment of people because of actual or presumed disabilities” (Campbell, 2009, p. 4). Goodley (2014) argues that ableism provides the “temperature and nutrients for disablism to grow” (p. xi). In education, disablism often manifests through the segregation of disabled students. In assessment research, disablism has been reported through, for example, negative staff and peer experiences toward disabled students (Hanafin et al., 2007; Marshak et al., 2010), and teachers’ unwillingness to provide accommodations even when they are officially granted (Brandt, 2011; Kendall, 2016; Nieminen, 2020; Ryan, 2007).

## Study aim

In this study, *I* *examine the underlying mechanisms of ableism and disablism in the assessment of student learning in higher education.* This work is based on 139 disabled students’ experiences of assessment, as collected through an open-ended survey in a Finnish university. Both assessment and assessment accommodations are concerned as they both play an important role in how disabled students come to understand themselves as certain kinds of learners in higher education. The responses are analyzed through a discursively oriented thematic analysis that identifies the mechanisms of normalization and division in students’ experiences. The aim is not only to reveal but to challenge the “‘neutral’ spaces of normalcy and the body politic” (Goodley, 2014, p. 25). My analysis provides critical tools to reconceptualize the “negative assessment experiences” of disabled students as reflections of systemic forms of exclusion and discrimination.

## Context

Finland enables an interesting and somewhat contradictory context for this study. At first glance, Finland seems to offer a fruitful context for the inclusion of disabled people in higher education. The Finnish welfare state provides free tertiary education for European Union students and provides compensation for the Finnish students’ living costs with a monthly grant. The legislation mandates reasonable adjustments in assessment for disabled students (Non-discrimination Act 124/2014, 15 §). Students need a documented reason, such as a diagnosis, to access such adjustments. However, policies differ between institutions in what counts as such a reason, and how adjustments are administered (Nieminen, 2021). Finnish universities do not have disability centers. Instead, each university has a disability liaison. Each university is required to conduct an equality plan based on the Non-discrimination Act.

While systematic data on disabled students has not been collected, recent surveys have shed light on the prevalence of disabilities in Finnish universities. In the Student Health Survey 2016 sampled at all Finnish higher education institutions (*N* = 4996 for academic universities), Kunttu and colleagues (2016) found that 6.5% of university students self-reported a “learning difficulty.” Dyslexia (4.4%) and ADHD/ADD (0.6%) were the most common of these. In a report concerning students with self-reported health problems and disabilities (sampled from all Finnish higher education universities, *N* = 5783, including universities of applied sciences), Korkeamäki and Vuorento (2021) noted that “learning disabilities” were reported by 4.8% (*N* = 103) of female and 3.8% (*N* = 48) of male university students, and “sensory impairments” by 1.2% (*N* = 24) female and 2.4% (*N* = 26) male university students (these numbers excluding students in universities of applied sciences). Of the factors hindering studying for all students with health issues or disabilities, the four most commonly reported were health-related issues, the difficulty and amount of studies, lacking motivation, and teaching arrangements. In all these categories, students reported significantly more issues than their peers without health issues or disabilities. These findings indicate that the number of disabled students in Finnish universities is rather small, and that they face barriers due to teaching arrangements.

No systematic studies have been conducted to map out the assessment culture in Finnish universities. However, my earlier document analysis of Finnish university policies and practices (Nieminen, 2021) revealed that assessment of disabled students is addressed as an exam-related matter. Inclusion was framed in policy documents as students’ right to participate in exams. Generally, assessment in Finnish universities is largely low stakes. Grades have relatively little impact on students’ future lives compared to countries where GPA has a stronger tie with future employment, and where students might compete over grades. Examinations can be taken multiple times. Assessment is typically criteria-based, as related to learning outcomes defined for courses, programs, and degrees. Teachers have vast academic freedom to choose their assessment practices.

Finnish universities have traditionally followed the Nordic welfare ideology. However, recent decades have seen neoliberal reforms aiming to increase their competitiveness in global education markets (FitzSimmons, 2015). According to FitzSimmons, the neoliberal shift toward market-driven ideologies has been rapid: this shift has had an impact on pedagogical practices through funding cuts, undervaluation of pedagogy, and overemphasis on certification and quick graduation.

## Methods

### Data collection

I conducted this study at a research-intensive university in Finland. To enable an institution-wide analysis, a digital survey to map out disabled students’ experiences of assessment was developed in Spring 2020. I collected pilot data (*N* = 25) in April–May 2020 to test whether the survey adequately captured the students’ rich conceptions and experiences related to assessment. Special attention was given to whether the survey mapped out not only experiences about exams but about other forms of assessment as well. Based on the comments by the students, the final survey was developed, and the dataset for this study was collected in November–December 2020. The data were collected in the context of the COVID-19 pandemic: while the survey was not designed to specifically capture the impact of the pandemic to assessment, this aspect was naturally reflected on the dataset. I developed the survey to enable a complex understanding of students’ experiences and conceptions of assessment and assessment accommodations (Appendix). For the purposes of this study, I only analyzed students’ *experiences*.

No official records could be retrieved specifically for those students who were officially granted assessment accommodations. I therefore had to identify participants from the whole student population. This was desirable also due to the high level of teacher autonomy. Especially in smaller courses, some teachers might provide assessment accommodations without students having to officially apply for them (Nieminen, 2020). Thus, the aim was to reach students who did not have an official approval for accommodations but had still used them. An invitation to participate in the survey was sent via email to all students (bachelor’s and master’s programmes) who were currently enrolled in the university and who had completed at least 30 study credits (The European Credit Transfer System). This resulted in 8733 students receiving the invitation. The email explained what assessment-related accommodations are and that anyone who had used them was welcome to participate. In addition, students who had reason to apply for accommodations but had not done so were also invited to participate. Participation in the survey was made possible via email or video call, and the participants were also able to submit their response as a separate file. However, no student used these possibilities.

### Participants

In total, 139 students responded to the survey. The basic demographic information is presented in Table 1.

**Table 1:** The study participants (*N* = 139)

Degree		Faculty				Gender		
Under-graduate	Post-graduate	Philosophy	Science and forestry	Health sciences	Social sciences and business	Female	Male	Other/I would rather not answer
69	70	58	10	13	58	104	26	9

Of the participants, 67 had officially applied for assessment accommodations, and 83 had used them. The students described in their own words why they were eligible for applying for accommodations; they were guided to share as much of their condition as they wanted to. The responses were loosely categorized to describe the dataset: disability (73), health (26), mental health (24), other (17), and I would rather not specify (8). Table 2 shows the accommodations used by the students:

**Table 2:** Accommodations used by the students (*N* = 83). Note that the same student might have used multiple accommodations

Extra time in tasks and exams	Alternative assessment format	Alternative exam arrangement	Flexibility with language	Supportive technology	Exemption from compulsory attendance
58	13	47	8	19	10

### Analysis

The analysis drew on the reflexive thematic analysis framework by Braun and Clarke (2020). This method offered tools for considering both students’ lived experiences through a data-driven approach and conceptual rigor through a further theory-driven analysis. The analysis consisted of six phases (Braun & Clarke, 2020), of which the first three comprised a data-driven part of the analysis, and the latter three the theory-driven part: (1) data familiarization; (2) systematic data coding; (3) generating initial themes from coded data; (4) developing and reviewing themes; (5) refining, defining, and naming themes; and (6) writing the report.

The data-driven part started with careful familiarization with the dataset. Throughout this phase, I read all responses by one student as a whole to capture a full sense of their experiences. The second data coding phase drew on in vivo coding by using students’ own words and utterances (Saldaña, 2016). Given that the data consisted of survey responses, this coding method ensured sensitivity concerning meaning and interpretation. In this phase, I restricted the dataset to codes that considered students’ *experiences*, restricting excerpts about, for example, students’ *conceptions* of assessment (if presented without a clear connection to experiences). In the third phase, I analyzed the coded dataset through a data-driven thematic analysis that considered themes as “patterns of shared meaning, united by a central concept or idea” (Braun & Clarke, 2020, p. 14). This phase provided knowledge about the prevalence and intensity of the constructed themes.

The main analysis was theory-driven. The initially themed dataset was re-interpreted (phase 4) and then revised (phase 5) through the theoretical frameworks of ableism and disablism. The thematic map that was constructed in the first phase was re-read by identifying the mechanisms of normalization and division. Phases 4 and 5 thus involved a discursively oriented analysis that understood the data excerpts as a part of broader discourses concerning assessment and inclusion. In the process, I formulated the data-driven themes to understand how ableism and disablism operated in assessment. The themes were not “separate” from each other, but many of the mechanisms for ableism and disablism overlapped and strengthened each other. Finally, writing the report was not seen as a separate process of the analysis but as an integral part of it. The next section does not showcase the final results of a “hidden” data analysis but lays open my thinking and interpretation in full, allowing criticism and contestation.

To ensure high quality of the analytic process as the sole author, I kept a digital logbook (with images, voice recordings, and text) throughout the analysis process. I drew on Lincoln and Guba’s (1985) concepts of credibility and dependability. Credibility aims for “truth,” which here is understood as a relational concept. The responses were analyzed with respect to being sensitive to the students’ experiences. The theoretical concepts guided the analysis and prevented “bias” which might result from inductive approaches. Dependability refers to consistency and contestability. This was achieved by a consistent use of theory, and through a large number of data excerpts to enable readers to critically contest the findings.

## Findings: unveiling ableism and disablism in assessment

Throughout the findings section, I refer to the students in brackets with gender-neutral pseudonyms and with students’ self-identified disabilities. First, I present a brief overview of the dataset. Even though the survey asked about students’ experiences of assessment in general, the findings largely concerned exams: exams characterized students’ assessment experiences. The students expressed their dissatisfaction with the overemphasis on exams and essays. Throughout the dataset, the students called for more student-centered and authentic assessment practices that would better align with their future profession, such as self- and peer-assessment and project-based assessment. When it comes to assessment accommodations, the students mostly perceived them as helpful: “Through accommodations, I have been able to show my real skills and abilities. That itself has been empowering” (Tyrni, dyslexia). Fifty-six students explicitly mentioned that accommodations have supported their learning and studying. The students with health-related issues in particular had mainly positive experiences of accommodations. In the following section, the constructed themes are presented (Table 3).

**Table 3:** Overview of the themes

Ableism	Disablism
**Themes:**	**Themes:**
• The dominant, normalizing gaze of exams	• Internalized ableism and stigmatization
• Holding disabled students responsible through assessment accommodations	• Denying accommodations or not organizing them properly
• Categorization	• Hassle and fight
• Marginalization	• Disclosure – again and again
• Dehumanization	• Opting out and failing
• The dominant role of language and text in assessment	
• Time management	

### Assessment and ableism

#### The dominant, normalizing gaze of exams

Exams were identified as a major mechanism for ableism through their normalizing gaze. As exams were designed for “the ideal, normal student,” they caused significant barriers for disabled students. Almost all the participants had negatively oriented exam-related experiences. Students named exams as a distressing factor that caused profound barriers:

Exams cause me a lot of stress. But I don’t want to go back to those distressing memories. I’ve noticed home exams don’t stress me as much as normal ones. I’m safe now. (Naava, ADHD)

I can’t cope with exams. There’s only a limited time and answers are written by hand. For me, the best assessment methods are learning tasks that you can do online and that give you enough time to complete them. I can complete them with my computer, at my own pace. There’s no panic or distressing feeling of having to escape the room. (Kuura, dyslexia)

In order to proceed in one’s studies, one needs to face multiple exams: an ideal student would be an efficient test-taker. Exams were thus identified as a major mechanism for framing disabilities as abnormal deficits. Many students described having found a discipline that best fits their personal needs and strengths. The issue was not that they would not have succeeded in their studies: the issue was exams. Aarre (ADHD, dyslexia), who excelled in computer science but was unable to succeed in exams, explained: “With my reading comprehension skills and with my slower pace, completing courses with exams is painful.”

Even though exams were widely criticized, only a handful of students suggested radical changes, such as doing away with them (such as Pihka who wrote “Let’s ditch traditional exams!”). This reflects the dominant role of exams in the assessment culture of this university. The students were well aware of the institutional constraints of assessment. For example, while Käpy (depression) wished for a more diverse “assessment menu,” Käpy understood that “if there are a lot of students, any other form of assessment [than exams] is time-consuming.”

#### Holding disabled students responsible through assessment accommodations

The students largely expressed their gratitude for assessment accommodations. Yet the analysis revealed how accommodations normalized disabled students to achieve just like “normal students,” rather than letting their unique personality be shown in assessment. The very underpinnings of the accommodation system held disabled students responsible for change. Exams caused barriers for students, and those barriers were then overcome through individual assessment accommodations. Disabled students were framed as the problem to be fixed, as opposed to the assessment itself. Kataja’s story shows how accommodations “fixed” Kataja’s challenges caused by dyslexia, while the original assessment culture remained unchanged:

I remember the moment in the exam hall when I first panicked. I wanted to escape the situation but I couldn’t move. This still brings tears to my eyes. When I found out about the possibility of accommodations, and when I finally got the permission, I cried out of relief. [– –] Thank you so much for assessment accommodations. I don’t know what I would do without them, whether I would even study here anymore. (Kataja, dyslexia)

The “accommodation menu” predominantly focused on exams in students’ experiences. Elo (dyslexia) criticized this and noted that “in an ideal situation my need for extra time would be considered during courses, not just in exams.” Ilves (dyslexia) had asked their department’s faculty about the types of support they could receive: “The message was that learning disabilities are addressed only through exam arrangements.” These experiences reflect not only how stable the existing menu of assessment practices and accommodations was, but also the crucial role of exams in constructing barriers and thus disabledness.

The students often called for support for managing deadlines and planning their studies. However, the assessment accommodation menu rarely addressed these needs. Tuovi’s experience shows how disabled students were held responsible for acquiring a diagnosis, for filing applications and even for caring about their teachers’ emotions:

The report from my study psychologist, that I had to get for my assessment accommodation application, stated that I should receive support to help me plan my schedule. This is especially important for longer tasks, to set my goals and reach them. But I haven’t been able to use this recommendation. I feel it’s tricky and awkward. This kind of requirement is probably rare and might confuse teachers. (Tuovi, learning disabilities)

The students hoped for proactive support, such as “automatically offering accommodations at each course” (Vuono, dyslexia). It was the students’ own responsibility to notice one’s need for support, apply for accommodations, and make sure they are properly administered. Many called for proactive screening procedures:

It was hard for me to recognize my ADHD for a long time. I wonder why students aren’t offered the possibility of a voluntary disability screening at the beginning of their studies. (Sävel, ADHD)

#### Categorization

The assessment accommodation system is based on the categorization of students’ conditions. Such categorization is precisely how dividing practices operate. The categorization process produced the categories of normal and abnormal, as the students understood themselves as “a person with an ADD brain” (Kuutti), a “different kind of a learner” (Vilja), or an “ADHD person” (Ulpu). These examples show how the accommodation system required students to understand themselves through cognitive and medical categories. While such categories might result in further care, support, and empowerment, ableism was identified in how students *had* to operate through these predetermined categories to access assessment accommodations: assessment further stabilized the pathological disability categories from each other and from “the normal student.”

The predetermined categorization system was unable to meet the hybridity of students’ needs and identities. For example, Pujo had a “long history with depression” and a physical condition that caused chronic pain. Pujo was a parent of a young child who was “lonely, living in a small town without friends.” Pujo was also struggling with “stressful fertility treatments,” and described themself as a perfectionist. None of these intersectional identities was taken into account by assessment accommodations: Pujo only received extra time for exams and essay submissions. Säde reported a similar experience:

My faculty has only granted me extra time for physical and online exams. For any other adjustments, I needed to apply for these directly from the teachers (e.g., more time for submissions), but they were never approved. Nowadays I don’t even bother asking anymore. (Säde, ADHD)

The assessment accommodations mostly made allowances only for psychologically and medically oriented conditions such as learning or physical disabilities and mental health issues. Sora’s experience is an apt example of this. Sora had divorced and been left alone with her children. Recently, a close family member had died from COVID-19. However, Sora had not applied for assessment accommodations: “I haven’t dared. First I’d need to ask for psychological help, but everything feels slower for me now. I need to concentrate on my well-being before completing courses. But because I haven’t applied, I might run out of time and lose my student grant. I try not to think about it.” Sora’s case demonstrates the concrete consequences of the categorization system: falling outside of its predetermined categories can be costly.

#### Marginalization

Through assessment accommodations students came to understand themselves as marginalized: the barely existing Others. For example, students frequently lamented the lack of available information on accommodations: “How do I even apply for assessment accommodations? Would I be eligible? Would it be possible to mention something about this to the students?” (Kuu). Often, students had not used assessment accommodations because they simply did not know they existed: “I never realized my neurological condition would qualify me for accommodations” (Siili). For some students, the survey itself was a “miniature intervention” that broke the mysticity around assessment accommodations: “After answering this survey I considered for the first time that I might benefit from assessment accommodations” (Talvi, dyslexia).

The students described an overall lack of representation of disabled people at their university. For instance, Valo (dyslexia) and Tuli (health-related issues) both stated that there should be more talk about assessment accommodations to raise awareness. Pääsky (health-related issues) wished for normalization of assessment accommodations:

Assessment accommodations should be made a normal thing, because now many people don’t want to apply for them for fear of getting labeled. At the beginning of each course, the teachers should mention the possibility of accommodations. At the moment it’s up to students to find the information and take care of their own accommodations. (Pääsky)

#### Dehumanization

The assessment accommodation process was defined as bureaucratic and dehumanizing. The same applied for exams. What the students wished for were *humane* assessment practices through which they could show their unique, personal human capabilities, such as face-to-face feedback, group discussions for peer feedback, and “interactive relationship with teachers, as it would be nice if teachers would know about my challenges for learning and would support me, and it would be nice to hear them ask how I’m doing with my tasks” (Siili). Syksy described the dehumanizing bureaucracy around assessment accommodations:

I’ve downloaded that document [application for accommodations] to my computer many times, but the bureaucracy and the officiality make me anxious. It’s somehow hard and distressing. (Syksy, mental health issues)

While the students described the assessment accommodation system as slow, inflexible, and dehumanizing, many reported a positive story about humane teachers showing their support. These stories, such as the one by Nietos below, showcase how individual teachers enabled students’ experiences of care and support. Yet, even in these examples, individual teachers provided these experiences, not the structural forms of support of the university.

It makes me feel grateful and even touched that this teacher seemed to sincerely care about my well-being. The teacher was ready to break the rules for me. That was the first time at university that I felt like the teacher really cared about their students. (Nietos, health-related issues)

#### The dominant role of visual assessment

Students largely described struggling with visual forms of assessment. Visual assessment divided students into the able and the disabled, even when text- and language-related skills were not learning objectives. As assessment was strongly text-based, issues arose for many students. Ableism was identified in how text-based assessment pointed its normalizing gaze especially toward disabled students. For example, Ahma (dyslexia) was taking an accounting course that needed to be completed through text-based essays. Ahma described being motivated to learn, but assessment caused them profound barriers. Ahma had to opt out: “I dropped out of the course and hoped there wouldn’t be any similar ones. The point of the course is to teach important skills related to accounting, calculations and interpretation of data. You can’t assess those skills only based on overly long essays, because producing them is especially hard for me.” Kuura (dyslexia) wished for portfolios that could encapsulate their continuous learning and development, and for learning material in audio format. Emi (dyslexia) stated that they could show their true skills through oral presentations and videos: “It would be awesome to get to do a video essay for some course. Editing might be a bit challenging, but so is writing for me.” Thus, to become included as “the ideal student,” one needed to be proficient at handling visual information, often within a certain time limit (e.g., exams). As disabled students could not meet this ideal, they were deemed unable to meet the academic standards, as measured through inaccessible forms of visual assessment.

#### Time management

Another mechanism for normalization, often taken for granted, was time. Time was a recurrent theme in the dataset. The students often defined themselves as someone *needing extra time*. Through normalizing assessment practices, they had come to understand themselves as too *slow* to be “the ideal student” who studied and graduated *quickly*. For instance, as Rousku (autism, depression) summed it up, “there are no assessment practices that don’t suit me, just ones I need more time with.” Drawing on Bennett and Burke (2018), time is conceptualized here as a relational, social phenomenon that determines “who is included and who is recognized as ‘capable’ in different higher education contexts” (p. 913). Managing disabled students’ time was, then, identified as a mechanism of ableism. Time management was framed as students’ personal responsibility rather than as a structural issue.

Assessment accommodations were needed to address the stress that time limits caused, but such “support” was not the support the students would have needed. Instead, the students largely wished for support for time management, goal setting, and study strategies to cope with the ableist time structures of assessment. Mesi blamed themself for being too “slow” in exams, even after receiving extra time:

Despite the extra time, I often experience the time pressure as distressing. Due to the limited time I have, I don't always have time to answer all my questions thoroughly, nor do I have time to show all my skills needed in the task, or revise my answers. (Mesi, dyslexia)

Pihka pleaded not to be viewed as “stupid” with respect to the dominant time structures. Through assessment, the students had come to understand “abledness” and “quickness” as inseparable:

Different kinds of learners should be considered. We are not stupid, we just need more time for reading and for completing tasks and exams. [– –] I have learned to survive and to use much more time for studying than everyone else. (Pihka, dyslexia)

The assessment accommodations process itself was time-consuming. As Tuovi put it: “It would be good to mention something about accommodations at the beginning of university so people who are eligible would realize to apply for them, as the process takes such a long time” (Tuovi, learning disabilities). This is another example of how disabled students’ time was managed: one of their responsibilities was to *wait*.

### Assessment and disablism

#### Stigma and internalized ableism

Disablism was identified in the outright discrimination that was connected to assessment accommodations. Even though assessment accommodations were perceived as helpful, the students also largely connected them with shame and embarrassment. Kide justified why they had not applied for accommodations: “There’s a fear of stigma around assessment accommodations.” Thus, assessment accommodations did not only exclude students socially and physically, but taught students to understand themselves as “the Other.” Assessment internalized ableism: this is *disablism* in action. Savu described the fear of stigma:

It feels like being labeled as ‘invalid’ and ‘disabled’ when you apply for help. Now that I’ve ended up studying at university, I feel that I just have to survive. I won’t be getting any support in future working life either. (Savu, dyslexia, mental health issues)

Some students reported that assessment accommodations were seen as unfair by others. Kiiski (dyslexia) stated: “Some fellow students are jealous about assessment accommodations. The common understanding is not that they offer similar chances for everyone, but that accommodations enable better chances.” Kuura (dyslexia) described stigmatizing, disablist actions by teachers:

Students with dyslexia start out hearing that they are a failure, stupid, and worthless. [– –] All by themselves, they learn to survive with the same requirements as everyone else to get what they want. When they finally build their identity and acknowledge their rights, they face mostly very cold attitudes as they cause extra effort for teachers. (Kuura)

Internalized ableism was identified as students understood themselves through their “deficits” as a “burden.” They had learned to understand themselves through stigmatizing practices as taking teachers’ resources—not as diverse students that enrich the university.

I feel embarrassed to ask examiners to take notice of me and arrange my own proctor and special room for exams. (Emi, dyslexia)

To me, my illness is a very sensitive topic - maybe I haven’t completely accepted it myself. I wouldn’t like to be a “burden” for anyone and cause extra work. (Nova, health-related issues)

#### Denying accommodations or not organizing them properly

Students had experiences of individual teachers denying access to assessment accommodations even when they were officially granted. This is an example of outright discrimination of disabled people in assessment. Karppi (ADHD, dyslexia) reported searching for help but not receiving answers from any of their teachers: “As I didn’t receive answers, I opted out and went to another course. I’ve never received help with my dyslexia.” Paiste (learning disabilities) described their teacher denying access to alternative assessment formats even when Paiste had an official permission for such alteration: “Later I found out that I absolutely should have been able to change the exam to an essay. That’s what it says in my accommodation decision.”

Some experiences of denial were so emotional that students had required external support. Kuutti’s (ADD, depression) teacher denied them access to extra time. After Kuutti showed the teacher their medical documents, the teacher asked whether Kuutti had thought about changing careers:

The teacher mentioned that at this point of my studies I should be able to stick to my schedules. [– –] I thought: ‘Is this it? Is there no place for me in this society, any career where I would fit, any job that I would be able to do? Should I just opt out and stop wasting tax money and bothering people?’ I avoided any further harm as I got support from other teachers and authorities. I think it’s clear that this teacher did not understand at all what ADD means. (Kuutti)

Sometimes teachers provided accommodations in a discriminatory way. Kurki (sensory sensitivity) was able to change their pair exam to an individual one, but still needed to complete the exam in the same space as everyone else. Ilves (dyslexia) reported:

Sometimes I’ve needed to complete my exam in a closet. Sometimes I arrive at the exam and the teacher has forgotten to arrange my separate exam room. (Ilves, dyslexia)

Some students were denied diverse accommodations as such accommodations did not fit the testing culture. This reflects the dominance of exams, but also how within such testing cultures, disabilities were framed as *resource-intensive* deficits:

I applied for diverse assessment accommodations based on a diagnosis from a psychologist. Most of them weren’t provided because they were too arduous for the examiners. (Lupiini, ADHD)

#### Hassle and fight

The students described being held responsible for the hassle of applying for assessment accommodations (searching for information, writing applications, informing teachers about their personal condition, ensuring that accommodations were provided…). Hassle was the main reason described by the students for not applying for accommodations: “It seems like a very arduous process,” Orion (dyslexia). Similarly, Vanamo (dyslexia) stated that accessing assessment accommodations was “made very complex.” Some students chose to underperform on some of their exams rather than apply for accommodations: “Before each exam you’d need to contact the examiner early and present documents about your dyslexia. This procedure causes me more trouble than what it’s worth.” Extra hassle was required of students who needed support exactly for that—managing complex tasks—which caused them significant barriers:

The reason why I’m eligible for assessment accommodations is also the reason why I haven’t applied for them. My ADD makes it hard for me to find information, and it’s hard to plan my time management. I don’t always remember everything or get things done. So I haven’t been able to apply for accommodations either. (Neva, ADD)

Sometimes, students described having to fight for their right to assessment accommodations: “As a mature student with ADHD, [– –] I will keep on fighting for this degree” (Viima, ADHD). Paiste (learning disabilities) described how they needed to fight for those assessment accommodations that were granted to them. This fight included the responsibility of educating teachers:

It would be good if teachers were aware of assessment accommodations without me, as a student, having to explain these practices to them. I’ve been in many situations where I’ve asked for assessment accommodations, and the teacher hasn’t known how they are arranged because they’ve never come across such a case. I shouldn’t need to do that as a student, to educate teachers about how the special room for exams works, or how to reserve extra time for an online exam [in the online system]. (Paiste)

#### Disclosure—again and again

Goode (2007) coined the term extravisibility to showcase how disabled students need to repeatedly and visibly disclose their condition to others. Similarly, this dataset contained multiple stories of the students needing to disclose their condition continuously during their studies: disclosure divided students from other students *over and over again*. For example, after receiving access to assessment accommodations, some students needed to show their medical documents to each teacher separately before each exam. Continuous disclosure was criticized extensively by the students. Kaneli (dyslexia) was instructed that they only needed to inform their teachers about their extra time allowance. However, in practice, some teachers still asked Kaneli for written proof of their diagnosis before providing the accommodations: “The procedure causes me more harm than good as my dyslexia doesn’t bother me that much.” Some teachers required disclosure that was in violation of students’ privacy:

Some teachers have asked about the reason for my accommodations, because apparently it affects their assessment, whether I have ‘cancer or dyslexia.’ These experiences have been extremely unpleasant. It’s been really draining having to explain to teachers that no, you can’t do that. (Peura, dyslexia, ADHD, autoimmune disease)

#### Opting out and failing

The assessment practices framed students as “abnormal” by dividing them from “other” students. Disablism was identified as such exclusion manifested in the students opting out of their courses or failing them. The discriminatory consequences of inaccessible assessment were evident in the students’ postponement of study plans: this affected their income and future plans. Lack of information was one reason for opting out: “I wish I would’ve known about the possibility to apply for accommodations earlier. I would have avoided failing a few courses for nothing” (Rousku, autism, depression). The experiences of Saukko and Tellus demonstrate how disabled students had been made to fail multiple times during their studies because of inaccessible assessment practices, which resulted in them pushing for their rights to equal opportunities.

For two years I’ve needed to complete this one course. No matter how many times I’ve asked, it hasn’t been possible to receive assessment accommodations. Now that the teaching staff has been mostly replaced, I finally received approval for accommodations and I can graduate with a bachelor’s degree. This is a great relief for me. (Saukko, anxiety disorder)

I won’t give up. I’m attempting an English course for the fifth time. It has now taken roughly 1300 hours of my time. I’m asking: Is this reasonable? Has my dyslexia been taken into account? (Tellus, dyslexia, hearing impairment)

## Discussion

In this study, I have analyzed the underlying mechanisms of ableism and disablism in the assessment of student learning, based on disabled students’ experiences. The findings showcase how test-driven assessment cultures set profound barriers for disabled students’ inclusion. In a way, there is nothing new under the sun: we already know that assessment disadvantages disabled students (e.g., Brandt, 2011; Fuller et al., 2004a, 2004b; Hanafin et al., 2007; Madriaga & Goodley, 2010; Nieminen, 2020; Riddell & Weedon, 2006). However, through critical use of theory, I have brought forth the politics of assessment by reframing students’ experiences as reflections of systemic forms of exclusion and discrimination. The main contribution of this study lies in the critical tools it enables for future research and practice.

The framework of ableism enabled me to understand how assessment favors normal and productive bodies and minds. Ableism was identified from the underlying structures of assessment such as time and visuality. Assessment accommodations divided disabled learners from *other students* physically and socially (Liasidou, 2014). Through the marginalizing and dehumanizing nature of the accommodation model, assessment actively framed disabled students as “the Other.” Based on these findings, it is argued that assessment is at the core of academic ableism. Assessment seeks and labels abnormality, enabling only predetermined, ableist ways of being and becoming a student. Thus, assessment sets substantial barriers to “celebrating diversity” in higher education. This was evident even in the low-stakes assessment context of Finland.

In contrast to ableism, whose normalizing gaze affects everyone, disablism only targets disabled people (Baglieri & Lalvani, 2019). Unveiling disablism in assessment is a crucial contribution: “inaccessible assessment design” and “negative experiences” as commonly reported in research were now framed in light of discrimination. Such discrimination directly contradicts attempts to include disabled students in higher education, being against moral imperatives and human rights (United Nations, 1948) and, in some contexts, against current legislation. The implication is simple: discrimination against disabled students through assessment needs to stop. As ableism provides the nutrients for disablism to grow (Goodley, 2014), it is important to disrupt ableism in assessment before outright disablism manifests. Designing assessment for “the normal and able student” builds the foundation for direct discrimination—especially in contexts where disabilities are predominantly stigmatized and understood as pathological deficits.

This study has particularly focused on exams. Closed-book exams have served universities for long, but they are unable to meet the needs of mass higher education in its quest for widened access and inclusion. I thus call for inclusive assessment initiatives to diversify the current repertoire of assessment practices. Otherwise, institutional resources may be wasted on *producing* barriers for learners through exams, and then overcoming those very barriers through accommodations. Presumably the accessibility issues related to exams are even more profound in high-stakes assessment cultures outside Finland. Yet, work on “inclusive assessment” will likely fail without understanding the politics of assessment. Testing promotes neoliberal ideas by rendering academic skills and knowledge into comparable market commodities—namely, scores and grades (Torrance, 2017). Moreover, testing upholds the competitive, meritocratic assessment system in higher education, which by default sets barriers for interdependence and communal approaches to assessment (Riddell & Weedon, 2006). Anti-ableist assessment then needs not only to challenge exams as an inaccessible assessment format but to disrupt the ableist ideologies of normality, objectivity, and productivity that testing promotes (Nieminen, 2022). Otherwise, the “elephant of traditional assessment” (Williams et al., 2014, p. 622) will remain in the room, hindering us from successfully implementing the existing knowledge about inclusive assessment design in practice.

These findings have shown how higher education institutions blame the victim, in Torrance’s (2017) words, by holding disabled students responsible for the failures of assessment. Disabled students are framed as a problem as they challenge the validity of assessment; they are then held responsible for finding solutions by applying for accommodations and by learning self-advocacy and time management skills (e.g., Slaughter et al., 2020). Such an emphasis on the responsibilization of individuals rather than on political structures reflects neoliberal ideologies (Dolmage, 2017; Goodley, 2014). The message for disabled students is that if they cannot be normalized for being able, productive, and competitive citizens, they cannot be *fully* included in academic communities. In the process, as seen in the findings, disabled students are taught to exclude themselves through internalized ableism. To address ableism and disablism in assessment, responsibilization needs to be directed at higher education institutions, as well as to policy makers. Furthermore, these findings have showcased how individualistic approaches to “inclusive assessment” are likely to remain performative and empty: they only provide a false sense of inclusion (Stentiford and Koutsouris, 2021). Social epistemologies and interdependence are needed to truly include disabled students in academic communities. As I have noted elsewhere (Nieminen, 2022), mass higher education will fail in its quest of inclusion as long as disabilities and diversity more broadly are not seen as enriching assessment but obscuring it.

This study has its limitations. While the survey study design enabled an institution-wide analysis, the data are limited in depth and size. Future studies should draw on deeper datasets (e.g., narrative interview data) (see Madriaga et al., 2010) and intersectional analyses. No health-related register data are available in Finland; this study has relied on students’ self-identified information. The study did not draw on participatory approaches, but the participating students’ voice was filtered by my own voice. Future research could involve students as co-researchers, perhaps through design-based approaches. Similarly, teachers could participate in future studies as co-researchers. Teacher perspectives could shed light on teacher agency in the questions of inclusive assessment, perhaps by analyzing how neoliberal and meritocratic ideologies of assessment restrict teachers’ inclusion work. Inclusivity from the viewpoint of teachers presents another important topic for future research: inclusive assessment needs to be inclusive for the diversity of teachers as well! Importantly, the study has been conducted in the low-stakes yet exam-driven assessment culture of Finland. Even in this context, assessment was built on ableist premises in students’ experiences. While similar findings have been reported elsewhere, one should note the contextual nuances while transferring these findings to other contexts. Finally, this study largely focused on exams and accompanied accommodations as they dominated students’ experiences of assessment. Future research could be conducted in contexts where assessment practices are more diverse (see Ashworth et al., 2010; Tai et al., 2021).

## Now what? Reframing assessment as a major catalyst for inclusion

As this study was undertaken, I found myself growing increasingly pessimistic. My original intention was not to write a cynical research article! Unfortunately, the students’ experiences revealed injustice in alarming ways. Yet, the frameworks of ableism and disablism are by no means meant to paralyze, but to move assessment research and practice forward: what is crucial is what happens next. I outline three key trajectories for future research and practice.

First, in Slee’s (2019) words, we need to *fail better* while striving for inclusive education. It is not enough anymore to report disabled students’ negative experiences of assessment. Critical approaches are needed to unpack *why* and *how* assessment has failed to meet its goals of learning and inclusion. I question the need for further inductive analyses of disabled students’ assessment experiences (e.g., Edwards et al., 2022; Hanafin et al., 2007; Slaughter et al., 2020) and instead challenge the research field to move further through theory-driven approaches. This study has enabled critically-oriented tools for such endeavors.

Second, I propose that *all* future inclusion work in higher education needs to address assessment. There is a lot we can learn from Barnes and colleagues (Barnes et al., 2000) who noted long ago that assessment is the engine of educational reforms: “Curricular reform undertaken without corresponding assessment reform appears unlikely to succeed” (p. 646). Thus, future work should place assessment at the very center of inclusion work. Anti-ableist work might need to start from reforming assessment given how profoundly assessment steers higher education toward neoliberal ideals. Similarly, future work on assessment cannot address student diversity as a separate “aspect.” In mass higher education, designing and reforming assessment needs to start from the very idea of student diversity. We cannot design assessment for “the ideal student” anymore: widening the narrow idea of normality and productivity that assessment often promotes is likely to support the learning and inclusion of all students. Now that the COVID-19 has already challenged us to rethink assessment, it is possible to seize the moment and harness assessment as a vehicle for celebrating diverse human capabilities (Nieminen, 2022).

What is needed is a reframing of “inclusive assessment” as a communal project between students, teachers, researchers, administrators, policy makers, and other crucial stakeholders. Higher education institutions play a major role in providing space for such work. No systemic change toward socially just futures succeeds without systemic approaches. Hearing the voice of disabled students themselves while transforming assessment is necessary. Participatory approaches will be highly useful for future research on inclusive and accessible assessment. Importantly, research communities can well be held responsible for providing useful knowledge about inclusive assessment. How communal approaches could challenge the deep individualization in assessment and grading offers a crucial reflection point for higher education in the post-pandemic world.

## Appendix. The questions on the survey.

1. Describe assessment practices in a university course during which assessment supported your learning and studying. Why did this kind of assessment support your learning?
2. Describe assessment practices on a university course during which assessment did not support your learning by not fitting your needs. Why were these assessment practices unfit for you?
3. How would you have changed the assessment practices as described in your earlier answer so that they would have fitted your needs better?
4. Share one memory or experience concerning assessment accommodations that is especially important or close to you, and that perhaps raises emotions. Why did you choose this specific experience?
5. Describe the university course of your dreams whose assessment practices you could choose yourself. Why would this kind of assessment support your learning and needs?
6. Which three assessment commands would you give to university teachers if you could change anything in assessment? Write these in imperative. For example, “get rid of all assessments” or “instead of grades, use only pass/fail”.
7. How would you develop assessment accommodations?
8. Finally, I would like to say that…

## Footnotes

1 ‘Disabled students’ is used rather than “students with disabilities” to highlight the active role of assessment in *disabling* students. The concept shifts the focus from the categorization of students to the disabling mechanisms of assessment. The study employs a broad definition of “disability” to consider various kinds of disabilities, illnesses, mental health issues, and impairments, and their intersections, while emphasizing the social, historical, and political underpinnings of what counts as “disability” and how “disabledness” is produced.

2 In fact, researchers have often preferred “test accommodations” as a term over “assessment accommodations", which underlines the dominant status of testing in higher education (e.g., Ketterlin-Geller & Johnstone, 2006; Slaughter et al., 2020; Weis & Beauchemin, 2020).
